# Role of maximum androgen blockade in advanced prostate cancer

**DOI:** 10.4103/0970-1591.45536

**Published:** 2009

**Authors:** Rajinikanth Ayyathurai, Rosely De Los Santos, Murugesan Manoharan

**Affiliations:** Department of Urology, University of Miami Miller School of Medicine, Miami, FL, USA

**Keywords:** Advanced prostate cancer, maximum androgen blockade, total androgen blockade

## Abstract

Androgen ablation is the mainstay treatment for advanced prostate cancer (PC). Researchers proposed that maximum androgen blockade (MAB) therapy with antiandrogen agent in combination with castration might result in a better outcome among patients with advanced PC. In the last two decades, numerous trials and pooled data analyses were conducted to optimize the role of MAB in the treatment of metastatic PC. Non-steroidal antiandrogens administered as part of MAB proved to have a small (3%) survival benefit, however, the magnitude of this difference is of questionable clinical significance. Available evidence suggests that MAB should not be routinely offered to patients with metastatic PC, however, it should remain a reasonable option when discussing management. The standard first line treatment should be a monotherapy, consisting of orchiectomy or LHRH agonist. MAB still has a role as a short-term therapy (2-4 weeks). The ongoing large sample population based prospective studies may add new dimensions in the use of MAB in treatment of the prostate cancer in future.

## INTRODUCTION

Prostate cancer (PC) is the most commonly diagnosed cancer affecting men after middle age. Currently PC is a significant health problem in most industrialized Western countries with a reported worldwide 5 year prevalence over 1.5 million. Based upon the data from the United States, for a 50 year-old man with a life expectancy of 25 years, there is a 2.9% risk of dying from PC. At diagnosis, 20-30% of men will present with advanced or metastatic disease. Of those, one fourth will die due to PC within 2 years. The mainstay of treatment for advanced PC is androgen ablation.[1] Investigators proposed that maximum androgen blockade (MAB) therapy with antiandrogen agent in combination with castration might result in a better outcome among patients with advanced PC.[2] Ongoing studies may find new areas in which use of MAB may be of substantial benefit.

## HISTORICAL OVERVIEW

Sixty years ago, Huggins and Hodges first showed the palliative benefit of androgen ablation in men with bone metastases.[3] The principle of this therapy is to inhibit the biosynthesis of androgens, the hormones responsible for PC cell growth. The original methods used to achieve androgen ablation were bilateral orchiectomy and oral estrogen. Subsequently Huggins and Scott reported a secondary benefit by surgical removal of adrenals to block further androgen production which was the first demonstration of maximal or total androgen blockade.[4] Since then the hypothetical benefits of MAB for advanced PC has been debated and underwent rigorous trials.

MAB hypothesized that removing all circulating androgens by blocking adrenal androgen in addition to inhibiting testicular androgen production might be beneficial to men with advanced prostate cancer. The concept of MAB was revisited with the invention of synthetic luteinizing hormone releasing hormone (LHRH) and antiandrogens.[5] Although less effective than LHRH analogues, the use of new generation antiandrogens also resulted in chemical castration by blocking the binding of dihydrotestosterone to androgen receptor in the nucleus of PC cells.

Years later, a large number of randomized controlled trials (RCT) have been conducted to evaluate the effectiveness of MAB as compared with castration alone.[6] Further meta-analyses of the trials showed a therapeutic advantage of MAB, however, uncertainty has existed about the magnitude of benefit with MAB.[7,8] In this article, we aim to review the available evidence on the role of MAB in the treatment of advanced PC.

## RANDOMIZED CLINICAL TRIALS

From 1980 to 1991, 39 prospective randomized controlled studies (RCT) were performed to compare castration alone versus some form of MAB.[7] These studies used either flutamide, nilutamide or cyproterone acetate as the antiandrogen combined with surgical castration or LHRH agonists. Of these, cyproterone acetate (CPA) is a steroidal antiandrogen and the other two are non-steroidal antiandrogens (NSAA). Though most trials did not provide evidence of survival benefit with MAB, three studies reported improved survival with MAB.[9–11]

Crawford *et al*. studied the benefit of adding flutamide (250 mg 3 times a day) to daily leuprolide therapy in 617 men with M1 stage PC. This cross over randomized trial reported that addition of flutamide resulted in a significantly longer progression free survival compared to placebo and leuprolide (*P*=0.03).[9] Dijkman *et al*. randomized 457 men with stage M1 PC to receive nilutamide (300 mg/day for one month followed by 150 mg/day) or placebo after bilateral orchiectomy. The nilutamide group showed significantly higher overall survival and longer median time to progression (21.2 versus 14.7 months; *P*=0.002).[11] Denis *et al*. randomized 327 men with stage M1 PC to receive bilateral orchiectomy or monthly depot goserelin acetate (3.6 mg) with flutamide (750 mg/day). This study reported MAB has significantly better results for duration of survival (*P* = 0.04), time to death due to malignant disease (*P* = 0.008), time to first progression (*P* = 0.009) and progression-free survival (*P* = 0.02) compared to orchiectomy alone. This study has shown 7 month survival in overall survival and 23% reduction in mortality.[10]

## META-ANALYSIS OF MAB

Diverse reports from the clinical trials raised a controversy on the benefit of MAB in advanced PC. This led to further systematic review and meta-analysis of the data pooled from the RCTs. We identified six distinctive meta-analyses from the English literature.[7,8,12–15] Both literature based meta-analyses and individual patient level meta-analyses were included for the review. Table 1 shows the descriptive details of each meta-analysis.

**Table 1:** Meta-analyses on maximum androgen blockade for advanced prostate cancer

Meta-Analysis (MA)	Bertagna *et al*.	Caubet *et al*.	Aronson *et al*.	Bennet *et al*.	PCTCG Trial	Schmitt *et al*.
Study year	1994	1997	1999	1999	2000	2003
MA type	IPL	LB	LB	LB	IPL	LB
*N*	1056	3427	n.a.	4128	8275	6320
RCTs	7	9	20	9	27	14
MAB arm	Ni	Fl/ Ni	Fl/ Ni/ CPA	Fl	Fl/ Ni/ CPA	Fl/ Ni
Control arm	Castration	Castration	Castration	Castration	Castration	Castration
% M1-PC	n.a.	57-100	93	98	88	96
OSS of MAB	16%	22%	13%	10%	3%	5%

IPL-Individual patient level data; LB-Literature based; PCTCG-Prostate cancer trialists’ collaborative group; MA-Meta-analysis; Ni-Nilutamide; Fl-Flutamide; CPA-Cyproterone acetate; M1-PC-Metastatic prostate cancer; MAB-Maximum androgen blockade; OSS-Overall survival, RCT-Randomized control trial; n.a.-Exact numbers not available.

### Results from meta-analyses

The first meta-analysis from individual patient level data was published in 1994 by Bertagna *et al*.[12] This analysis included data from 7 RCTs of MAB with nilutamide. This analysis reported that the odds of disease progression was significantly reduced in the group of patients treated with nilutamide (OR 0.84, *P*=0.05). An abstract published by Debruyne *et al*. in 1996 updated the follow-up and survival.[16] This follow-up report concluded that MAB with nilutamide was associated with a 16% reduction in mortality as compared with castration alone (OR= 0.84; 95% CI 0.71-0.99; *P*=0.038).

The second meta-analysis was reported by Caubet *et al*. in 1997 comparing treatment with NSAA plus either LHRH or orchiectomy versus treatment with LHRH or orchiectomy alone.[13] The conclusion of this analysis stated that the relative risk (RR) of overall survival of the MAB group was 0.78 (95% CI 0.67- 0.90) by reconstructing an annual life table from geographical presentations of survival distributions and fitting discrete proportional hazard models and 0.84 (95% CI 0.51- 0.8) when hazard ratios were derived from reported P values and number of deaths. In all, 22% reduction in mortality was reported in patients treated with NSAA compared to castration alone.[13]

A large literature based meta-analysis was conducted for Agency for Health Care Policy and Research (AHCPR) by Aronson *et al*. in 1999. This analysis comprised of 20 MAB trials including NSAA and steroidal antiandrogens in the MAB arm. In addition to overall mortality, many other aspects such as data on disease progression, quality of life, and adverse effects were also analyzed. They concluded that the overall mortality at 2 years was not statistically different from the castration alone group. At 5 years the overall mortality was statistically significant with the MAB group (HR=0.87; 95% CI 0.81-0.94). However, the authors added that the magnitude of this difference is of questionable clinical significance because the results were based upon half of the patients that contributed to 2 year analysis. Subgroup analyses on type of antiandrogen, method of androgen suppression and stage of disease did not show any difference in survival.

In 1999, Bennet *et al*. conducted a meta-analysis of all the 9 RCTs comparing treatment with flutamide plus castration with castration alone. Pooled estimates demonstrated a 10% improvement in overall survival with flutamide as MAB therapy (RR =0.90, 95% CI 0079-1.00).[8]

In 1995, the Prostate Cancer Trialists' Collaborative Group (PCTCG) published their first individual patient level meta-analysis comprising 5170 patients and 22 RC- MAB trials.[6] The limitations of this analysis were the absence of precise inclusion criteria and end points other than overall mortality was not analyzed. Subsequently this report was updated in the year 2000 by another individual patient level meta-analysis which reviewed a total of 27 RCT, including those used steroidal antiandrogen in MAB arm.[7] The pooled data analysis of 8275 patients in this report represents the most comprehensive quantitative analysis of MAB trials. The updated analysis (2000) from PCTCG reported 70.4% mortality in the MAB group and 72.4% mortality in the group received castration alone. At 5 years, the reported survival was 25.4% with MAB and 23.6% with castration alone, suggesting an absolute survival difference of 2% (HR=0.96; 95% CI 0.91- 1.01; *P*=0.11). In subgroup analysis, a small but statistically significant survival benefit was observed for MAB with flutamide (HR=0.92; 95% CI 0.86-0.98; *P*=0.02), and a similar but non-significant result was noted for nilutamide. The results for CPA, which comprised of 20% of the study population, was unfavorable for MAB (5-year survival 15.4% MAB versus 18.1% castration alone (HR=1.13; 95% CI: 1.01 - 1.25; *P*=0.04). The updated report concluded as treatment with MAB containing NSAA increased 5 year survival over castration by 3% (27.6% versus 24.7%; SE 1.3; logrank 2p=0.005).[7]

A recent meta-analysis on RCTs comparing castration with MAB using NSAA was conducted for the Cochrane Prostatic Diseases and Urologic Cancers Group by Schmitt *et al*. in the year 2003.[14] The pooled OR for overall survival with MAB at 5 years was better than castration alone (OR=1.29; 95% CI 1.11-1.50; *P*=0.0009).

## DISCUSSION

In the last two decades numerous trials and pooled data analyses were conducted to optimize the role of MAB in the treatment of metastatic PC.[17] Although extensive work has been done, clear guidelines were not reached because of the diverse results from the published reports.[18] This article reviews the available evidence on MAB therapy for metastatic PC and the current recommendations.

Reports from meta-Analysis are the best form of evidence currently available for review. Authors chose six meta-analytical reports which can be broadly divided into individual patient level data analytical reports and literature based analytical reviews.[7,8,12–15] Generally patient level data are felt to be the gold standard for evaluating data from several phase III trials compared to literature based reviews.[8]

The individual patient level PCTCG meta-analysis is the most popular and widely accepted evidence available to date.[7] This analysis answered two critical questions. Firstly, compared to castration alone, MAB with the steroidal anti-androgen CPA was associated with 3% increased risk of death. Secondly, it showed a 3% survival benefit at 5 years in patients receiving MAB with NSAA compared to castration alone.

However, Chodak *et al*. critically reviewed and raised a series of concerns on the reliability of the findings from the above meta-analysis.[18] The major failing of this study was the premise that all the combinations of castration and antiandrogens are equal. From the published RCT reports, it is evident that not all antiandrogens are same when comparing against castration alone. Accounting these differences studies using various antiandrogens should not have combined together in the meta-analysis. Further exploratory analysis by Chodak *et al*. showed that the study arms utilizing flutamide resulted in an inferior survival compared with bicalutamide (P=0.047). Moreover, the combination of flutamide with leuprolide resulted in significantly poor outcome compared to other study arms (*P*=0.008). These differences were not addressed in the study. Other concerns were assuming that the effect of MAB would be similar for men with minimal versus extensive metastases and the duration of MAB treatment. Most of the MAB trials were conducted during the pre-PSA time and continued antiandrogen therapy might have had adverse effect on the disease control. Moreover, during these trials the ideal duration of MAB to achieve optimal results were not known. Other studies have shown the difference in outcome when MAB was discontinued earlier than expected due to adverse effects.[19]

The literature based review by Aronson *et al*. was well conducted and includes clear inclusion criteria as well as publication bias.[15] This report answered few fundamental questions. They reported that survival after treatment with an LHRH agonist is equivalent to survival after orchiectomy and confirmed that all available LHRH agonists are equally effective. This report showed a small but statistically significant survival difference at 5 years among patients receiving MAB compared to castration alone.

### Recent developments

Usami *et al*. published a report on comparing MAB with bicalutamide 80 mg combined with LHRH versus LHRH alone. This phase III, double blind RCT showed favorable results for time to disease progression and time to treatment failure, however, the interim report on overall survival was not statistically significant.[20] In the recent years, the Japanese Urological Society is revisiting the role of MAB in locally advanced and metastatic PC. A latest retrospective report on 628 locally advanced PC, 63.5% were treated with MAB. This study also addressed the reduction of quality of life during therapy.[21] A large longitudinal observational survey report (*n*=26,272) published by the Japan study group for prostate cancer revealed 59% patients started on hormone therapy between January 2001 and December 2003 chose to receive MAB. The report highlighted that MAB therapy was often selected for high-risk patients. This study is ongoing and the long term results waited with much expectation.[22] Assessment of the cost-effectiveness of MAB has recently been studied in Japanese men with advanced prostate cancer. Much value in administering bicalutamide in these patients was found suggesting that it is a highly cost-effective therapy.[23]

Data recently published described neoadjuvant chemotherapy combined with MAB as a possible therapy for patients undergoing radical prostatectomy for clinically T3/T4 prostate cancer.[24] Nishimura *et al*. evaluated flutamide as second line therapy for hormone-refractory prostate cancer; response in patients was defined as a larger than 50% decrease in PSA levels at the beginning of therapy.[25] Flutamide proved to be an effective option in these patients. These studies both take a distinct look at MAB; further analyses may be needed to determine if this might represent a new role for MAB.

In summary, MAB utilizing CPA is adversely affecting the survival in patients with metastatic PC hence not recommended.[7] NSAA administered as part of MAB proved to have a small (3%) but statistically significant survival benefit, however, the magnitude of this difference is of questionable clinical significance.[7,15] Though data available on adverse effects and quality of life are limited, treatment with MAB suggests increased adverse effects and a possible decline in quality of life.[17] Available evidence suggests that MAB should not be routinely offered to patients with metastatic PC, however it should remain a reasonable option when discussing management. The standard first line treatment should be a monotherapy, consisting of orchiectomy or LHRH agonist. MAB still has a role as a short term therapy (2-4 weeks) in preventing testosterone flare while initiating medical castration with LHRH agonist.[26]

There is a continuous endeavor to re-examine the concept of MAB and new findings are being reported through the last few decades. The ongoing large sample population based prospective studies may add new dimensions in the use of MAB in treatment of the prostate cancer. However, current evidence displays limited therapeutic role for MAB in the treatment of advanced prostate cancer. New studies possibly will demonstrate a role for MAB in the near future such as in neoadjuvant therapy and as second line therapy for advance prostate cancer.

In addition, therapy should be further centered on maintaining significant quality of life, as well as cost-effectiveness of treatment. MAB therapy should be reevaluated with further trails undergone to specifically address these factors.
